# A System-Level Perspective on Epstein–Barr Virus Persistence: The Partial Lytic Reactivation

**DOI:** 10.3390/ijms27073337

**Published:** 2026-04-07

**Authors:** Krzysztof Piotr Michalak, Wojciech Adamski

**Affiliations:** Laboratory of Vision Science and Optometry, Physics and Astronomy Faculty, Adam Mickiewicz University of Poznań, Uniwersytetu Poznańskiego Street 2, 61-614 Poznań, Poland

**Keywords:** abortive lytic reactivation, viral latency, chronic inflammation, immune exhaustion, autoimmune disease, Epstein–Barr virus-associated disorders

## Abstract

Epstein–Barr virus (EBV) establishes lifelong infection in most humans, yet its biology in immunocompetent hosts is commonly framed as a binary alternation between latency and productive lytic replication. Accumulating molecular and single-cell evidence challenges this view, indicating that EBV frequently enters abortive forms of lytic reactivation that do not culminate in virion production. Here, we propose a conceptual framework in which EBV persistence is governed by feedback-regulated interactions and permissive conditions for reactivation rather than a strictly sequential life cycle. Immediate-early and early gene expression can be repeatedly induced by inflammatory signaling, cellular stress, and epigenetic changes. However, progression to viral DNA replication represents a highly functional barrier that likely requires the coordinated convergence of multiple viral and host conditions. Failure to reach this threshold arrests reactivation before late gene expression, generating a stable partial lytic state characterized by sustained immunomodulatory viral protein expression without the production of infectious particles. Immune surveillance reinforces this bottleneck by eliminating cells undergoing full lytic replication while sparing those stalled in early phases. We argue that EBV persistence reflects a dynamic equilibrium shaped by regulatory interactions between viral gene expression and host immunity, with implications for biomarker interpretation and therapeutic strategies in chronic inflammatory and autoimmune disease.

## 1. Introduction

The Epstein–Barr virus (EBV) is one of the most widespread human viruses and has been implicated in a broad spectrum of clinical manifestations across diverse patient populations. Despite its near-universal prevalence, modern medicine remains limited in its ability to accurately diagnose ongoing EBV activity or to establish clear causal links between viral reactivation and many associated diseases, particularly those with mild, fluctuating, or chronic courses. Epidemiological and experimental studies have reported associations between EBV and multiple sclerosis [1,2,3,4], rheumatoid arthritis [5,6,7], Sjogren’s syndrome [5,7], systemic lupus erythematosus [7,8], fibromyalgia [9,10], Hashimoto thyroiditis [11,12,13] and hemophagocytic lymphohistiocytosis [14,15]. EBV is also reported to be one of the viral causes of acute uveitis and acute retinal necrosis [16,17,18]. While the mechanisms linking EBV to severe clinical outcomes such as chronic active EBV disease (CAEBV) and EBV-associated malignancies are increasingly well characterized, the biological significance of milder, transient viral reactivations and low-level chronic activation remains poorly understood. Population studies suggest that this virus is detected in plasma in a transient or chronic state of excitation in a few percent of the population [19,20,21,22] and this part of population is the main current challenge for medicine and the main point of interest in the current work. In particular, it is unclear how such forms of EBV activity might persist despite immune surveillance and contribute to long-term immune dysregulation and autoimmune pathology.

This review examines the emerging concept of partial (abortive) viral reactivation as a key mechanism underlying chronic EBV persistence. Such states are characterized by incomplete immune clearance accompanied by substantial alterations in intracellular metabolism and immune signaling, without progression to full productive infection. The focus of this review is on partial lytic reactivation as a frequent yet underappreciated mode of EBV activity that functionally bridges latency and productive replication, providing a conceptual framework for understanding how EBV can remain biologically active while largely escaping conventional diagnostic detection. In this article, we use the term partial lytic reactivation to emphasize that this state represents a regulated and recurrent configuration rather than a failed lytic attempt.

Although abortive lytic reactivation has been previously described, it is typically treated as a transient or incomplete phenomenon rather than a central feature of EBV biology. Here, we propose a system-level perspective in which partial lytic reactivation constitutes a predominant and dynamically regulated state that links latency and productive replication. Within this framework, EBV persistence is viewed not as a binary switch but as a continuum of states shaped by interactions between viral gene expression programs, host immune responses, and cellular metabolic conditions. This conceptual framework provides a unifying interpretation of chronic low-level viral activity and its potential contribution to immune dysregulation and inflammatory or autoimmune diseases.

Importantly, this framework generates specific testable predictions. It suggests that a significant proportion of EBV-infected cells in vivo may reside in partial lytic states characterized by early lytic gene expression without full virion production. It further implies that such abortive activity may play a disproportionately important role in sustaining viral persistence and modulating host immune responses. Finally, it indicates that the detection of early lytic transcripts, rather than viral DNA alone, may provide a more sensitive approach for identifying subclinical EBV activity. This conceptual framework also provides a basis for future quantitative and mathematical modeling of EBV persistence dynamics. The interactions between viral gene expression states, host immune responses, and metabolic conditions described here can be naturally formalized using systems biology approaches, including differential equation-based models of state transitions.

These considerations also have important implications for current therapeutic strategies. In particular, approaches based on the “kick and kill” paradigm, which aim to induce lytic reactivation for subsequent elimination of infected cells, may require reconsideration within this framework. If lytic induction frequently results in abortive rather than fully productive cycles, then activation of immediate-early or early lytic genes alone may be insufficient to achieve effective clearance. Instead, therapeutic efficacy may depend on the ability to drive infected cells beyond key progression checkpoints toward fully productive lytic states, or alternatively, to suppress chronic partial lytic activity and returning the cell to the latent state.

### The Spread of the Virus in the Human Body

Many studies indicate that EBV DNA can be detected in individuals’ blood or PBMCs (peripheral blood mononuclear cells), with asymptomatic infection in 90–95% of the human population [23,24]. On the other hand, severe cases of chronic EBV infection (chronic active EBV, CAEBV), with large amounts of EBV DNA detected in patients’ plasma, leading to serious complications and even death, are relatively rare [25,26]. Tumors induced by the presence of this virus in tissues are also detected [27,28]. It should be assumed that between these two extreme cases there is a whole range of intermediate cases in which the activation of the virus is only temporary or chronic activation remains at a chronically low level, causing only minor clinical symptoms and slight deviations in laboratory tests. As mentioned earlier, EBV is detected in plasma in a transient or chronic state of excitation in a few percent of the population [19,20,21,22]. It is necessary to organize knowledge on this subject in order to improve the medical community’s understanding of EBV as a potential cause of non-specific symptoms in patients, as well as the mechanisms for detecting these conditions and the correct interpretation of available laboratory tests. Understanding the numerous molecular mechanisms of immune evasion and chronicity of the virus will also give an idea of the seriousness of the problem and the need to seek appropriate diagnostic and treatment strategies tailored to the molecular disturbances generated by the virus in cells.

## 2. Phases of Virus Activation

In most immunocompetent individuals, viral DNA resides in B lymphocytes and epithelial cells [29]. The viral DNA level is maintained at a very low level of 1–50 infected leukocytes per 1 million [30], consistent with an average of 1–30 EBV DNA copies per million leukocytes, where it can be detected by PCR testing of whole blood or PBMCs. At this level, the virus does not essentially induce transcription of its proteins, so there are no significant molecular disturbances or dysregulation of cell metabolism resulting from such latent presence. However, during periods of fatigue, stress or decreased immunity, the lytic phase of reactivation is periodically activated and the transcription of its own proteins begins, leading first to the preparation of the cell’s transcriptional apparatus for the production of its own virions and then to their production [31,32,33]. In the process of lytic reactivation, four phases are typically distinguished: the latent phase (Lat) the immediate-early phase (IE), the early phase (E) and the late phase (L) [34,35]. Several studies suggest, however, that viral DNA replication represents a discrete regulatory checkpoint between the early and late phases, functionally separating transcriptional activation from productive replication [36].

### 2.1. Latency Programs (Lat)

**Figure 1:**
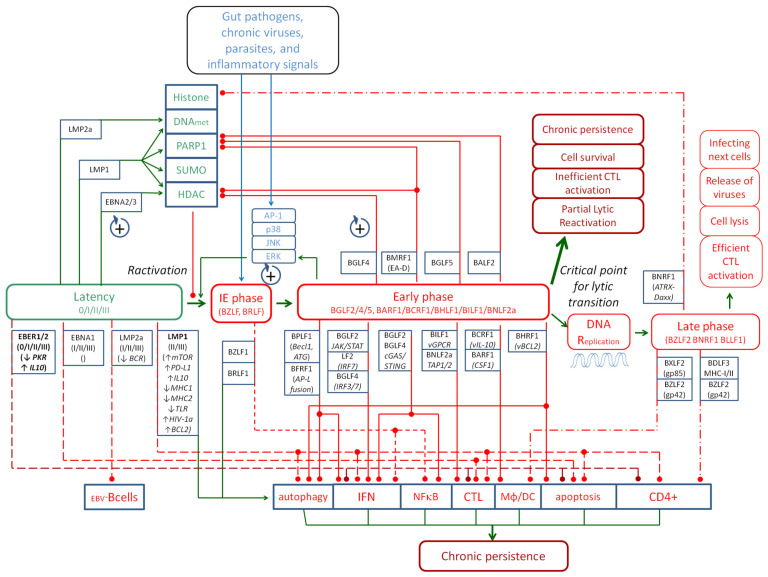
Schematic overview of Epstein–Barr virus (EBV) infection states and regulatory checkpoints. EBV persists in latency programs (latency 0/I/II/III), characterized by restricted viral gene expression including *EBNA1* (Epstein–Barr nuclear antigen 1), *LMP1/2A* (latent membrane proteins 1 and 2A), and *EBER1/2* (EBV-encoded small RNAs). Transition to the immediate-early (IE) phase is driven by *BZLF1* (Zta) and *BRLF1* (Rta), which initiate early (E) gene expression (e.g., *BGLF2/4/5*, *BMRF1*, *BALF2*, and *BNLF2a*). Early proteins modulate host antiviral pathways, including interferon (IFN) signaling (IRF3/7, JAK/STAT), antigen presentation (TAP1/2, MHC-I/II), autophagy (ATG, Beclin1), apoptosis (BHRF1), and innate sensing (cGAS/STING). NF-κB (nuclear factor kappa B) and MAPK (mitogen-activated protein kinase) pathways (p38, JNK, and ERK), and AP-1 (activator protein 1) facilitate the LAT → IE transition under inflammatory conditions. Epigenetic regulators (HDAC, SUMO, and PARP1) constrain viral promoter accessibility. Initiation of viral DNA replication represents a critical checkpoint separating partial from productive infection. Failure to progress to the late (L) phase (e.g., BZLF2, BNRF1, and BLLF1 production) results in partial lytic reactivation, characterized by IE/E expression without virion release. Limited late-phase progression enables the infection of new cells, while efficient cytotoxic T lymphocyte (CTL) responses restrict full lytic replication. The model depicts chronic persistence as a dynamic equilibrium between latency, partial lytic activation, and immune selection. For references—see Table 1. ⊕—positive couplings stabilizing Latency or Early phase state. Green lines with arrow—activation, red lines with dot—inhibition.

During latency, EBV exists as circular episomal DNA in the host nucleus, expressing a very limited set of latent genes, if any [37]. The four types of latent phase, 0, I, II and III, are described according to the expression of LMP1, LMP2a, EBNA1 and EBER1/2 [38,39]. Attention should be paid to EBER1/2, which is active in each of these phases and exhibits its immune-suppressing effects [40] (Figure 1).

Four major types of latency reflect both the cellular context of infection and the degree of immune surveillance. Latency I is marked by the expression of EBNA1, together with EBERs and viral microRNAs, supporting episomal maintenance while minimizing immune recognition [37,41]. Latency II includes EBNA1, LMP1, and LMP2A/2B, enabling modulation of the cellular signaling pathways involved in survival, differentiation, and immune evasion, and is commonly observed in epithelial malignancies [42,43,44,45]. Latency III represents the most transcriptionally active program, with expression of all EBNAs and latent membrane proteins, driving strong B cell activation and proliferation, as seen in lymphoproliferative disorders [37,46]. The establishment of these latency types is shaped by the host cell differentiation state, immune pressure, and epigenetic regulation of the viral genome. Importantly, latent proteins not only sustain viral persistence but also actively suppress lytic reactivation by inhibiting immediate-early gene expression and interferon-mediated antiviral responses [47,48,49,50,51,52,53]. This repression of lytic entry favors long-term survival of infected cells and creates conditions that promote genomic instability, chronic signaling activation, and resistance to apoptosis. As a consequence, latent EBV infection provides a molecular environment conducive to cellular transformation and contributes directly to EBV-associated oncogenesis.

**Table 1:** The effect of viral proteins on the immune system. Weakened virus control at the extracellular and intracellular levels facilitates the onset of chronic partial lytic infection. Arrows indicate the direction of regulation: ↑—activation or upregulation; ↓—inhibition, degradation or functional downregulation.

Inhibited Function	Viral Protein	Description	Ref.
Autophagy modulation	LMP1 (Lat)	Activates early stages, inhibits late stages of autophagy	[42,54,55,56,57,58,59]
	LMP2a (Lat)	↑ vPI3K/AKT/mTOR	[60,61]
	BZLF1 (IE)	Initial autophagy activation	[62,63]
	BHRF1 (E)	vBCL-2, inhibiting autophagy, activating mitophagy	[64]
	BPLF1 (E)	Inhibition by SQSTM1/p62	[65]
Interferon inhibition	EBER1/2 (Lat)	↓ PKR	[66]
	LMP1 (Lat)	↓ Tyk2	[67]
	BRLF1 (IE)	↓ IRF3/7	[68]
	BZLF1 (IE)	↓ IRF7	[69]
	BPLF1 (E)	(Deubiquitinase) ↓ TLR (TRAF6/NEMO/IKK) and RIG-I–TRIM25 axis	[70,71,72]
	BGLF2 (E)	SHP1 phosphatase recruitment, STAT2 degradation, ↓ Tyk2, STAT1, and STAT3 phosphorylation	[73,74]
	LF2 (E)	↓ IRF7	[75]
	BGLF4 (E)	↓ IRF3	[76]
	BHRF1 (E)	↓ vBCL2, mitophagy	[64]
	BFRF1 (E)	↓ IRF3	[77]
	BGLF5 (E)	Degradation TLR2/9	[78,79]
NFkB inhibition	EBNA1 (Lat)	Inhibition of IKK phosphorylation	[80]
	BGLF2 (E)	Inhibition of p65 Ser536 phosphorylation	[81]
	BZLF1 (IE)	TNFa/IFNg inhibitor	[82]
	BGLF4 (E)	UXT phosphorylation	[83]
	BPLF1 (E)	Deubiquitines TRAF6	[71]
CTL inhibition	EBNA1 (Lat)	NK cells inhibition	[77]
	LMP2a (Lat)	↓ CD8^+^	[84]
	LMP1 (Lat)	Inhibition of T cell proliferation and NK cytotoxicity	[85]
	EBER1/2 (Lat)	Inducing IL-10	[86]
	BCRF1 (E)	vIL-10	[87]
	BNLF2a (E)	↓ TAP1/2	[87]
Macrophages/DC	BCRF1 (L)	vIL-10	[87]
	BARF1 (E)	Binds CSF1, inhibits CSF1R (CD115) activation	[88]
	BZLF2-BXLF2 (L)	GM-CSF, IL4	[89]
Apoptosis inhibition	LMP1 (Lat)	RIPK1/3 ubiquitination. NF-κB activation, increasing MDM2 binding to p53, p53 ubiquitination and degradation, Bcl-2 expression enhancement	[90,91]
	EBER1/2 (Lat)	IFNα and GAS2 inhibition, BCL-2 activation	[92,93]
	LMP2a (Lat)	BCR inhibition	[94]
	BHRF1 (E)	vBCL2	[64]
CD4^+^ inhibition	EBER1/2 (Lat)	Inhibits IL12 and peptide presentation on HLA-II	[95]
	LMP1 (Lat)	Enhances PD-L1 level	[96]
	BZLF2 (L)	gp42, blocks antigen presentation on MHC-II	[97,98]
	BDLF3 (L)	Reduces expression of MHC-I/II	[99]

### 2.2. Immediate-Early Phase (IE)

Upon exposure to appropriate stimuli (e.g., B cell activation, chemical inducers, and epigenetic modifiers) [37], transcription of the viral IE-phase genes *BZLF1* and *BRLF1* is induced. Possible transcriptional activators include (but are not limited to) CREB, AP-1, HIF, ROS, PI3K/AKT and mTORC2. A detailed analysis of multiple factors that stimulate and inhibit excitation is analyzed in Wang’s publication [33]. However, for them to work, it is first necessary to relax the chromatin and remove factors blocking access to the promoters of these two genes, such as HDAC (histone deacetylase), PARP1 (poly(ADP-ribose) polymerase 1), SUMOilation and histone methylation, which permanently and strongly block the access of activating factors to DNA [34,47,100,101,102,103,104], so that reactivation, despite the presence of the virus in 95% of the population, is a rare phenomenon. Latent phase proteins also perform autoinhibition to prevent the virus from being activated too frequently [47,48,49,50,51,53]. Altogether, a broad array of factors can influence the initiation of this process and it undergoes a subtle equilibrium between inducing and silencing lytic induction [37]. Understanding this subtle balance is very important for the development of strategies preventing this event and silencing it in case of its induction.

The first viral proteins produced in this phase from *BZLF1* and *BRLF1* genes are Zta (ZEBRA) and Rta, which act as transcriptional activators of early (E) lytic genes and remodel viral and host chromatin to favor replication [37]. By expressing Zta and Rta, EBV primes the host cell and its own genome for DNA replication, early gene expression, and eventually virion production [37,105,106,107]. These proteins also initiate the inhibition of natural immunologic resistance in order to facilitate further stages of viral development. The exemplary activities are autophagy modulation [62,63] and the inhibition of interferons [68,69] and NF-κB [82] production.

### 2.3. Early Phase

In the early (E) phase, the virus prepares the replication apparatus and cellular environment for mass replication of the viral genome before structural proteins (late genes) are synthesized [108,109]. Early genes are defined as viral genes transcribed before viral DNA replication but after IE gene expression [109]. The early proteins produced include DNA replication enzymes (e.g., viral DNA polymerase and oriLyt replication proteins), viral kinases, host environment modifiers, cell metabolism modifiers, and proteins that help remove epigenetic or immunological barriers [34,109]. Examples of early phase proteins include BALF2 [110,111], BARF1 [88], BCRF1 [87], BFRF1 [77], BGLF2/4/5 [73,74,76,78,79], BHRF1 [64], BILF1 [112], BMRF1 [113,114], BNLF2a [87,115,116], BPLF1 [71], and LF2 [75]. All of these appear in the early phase and prepare the cell for, among other things, full virion production [109]. Early gene promoters often contain motifs for IE genes (*BZLF1* and *BRLF1*), which means that IE products activate early genes directly or indirectly [108]. E-phase proteins are also involved in numerous positive feedback loops with BRLF1 and BZLF1 to amplify reactivation [33,117] (Figure 1). The production of early phase proteins results in epigenetic changes like histone acetylation (DNA unwinding) [118,119], dePARylation [110,111,118,120,121] and changes in the activity of cellular pathways. The exemplary activated pathways are p38 [74,122], JNK (c-Jun N-terminal kinase) [74,123], AP-1 (activator protein 1) [124] and DDR (DNA damage response) [125]. However, when analyzing the links between the activation of the lytic phase and individual signaling pathways, it is important to note which of the many pathways are activators of the lytic phase and which pathways are modulated by the activities of proteins in this phase. These couplings can be positive, amplifying the lytic phase, or they can be negative, attenuating it. In the case of MAPK (p38, JNK, and ERK (extracellular signal-regulated kinase)) and PI3K (phosphoinositide 3-kinase) pathways, these couplings are positive, contributing in an additional way to amplify activation induction. A summary of the activation of the lytic phase by individual pathways is presented by Hui et al. [126]. The herbal strategies to treat EBV activation based on the herbal inhibition of lytic reactivation are presented by Li et al. [127].

Early gene products prepare the viral genome for replication: they activate replication origins (oriLyt), participate in the formation of replicons [128], support the release of viral DNA from chromatin, and modify the host cell environment to ensure access to nucleotides [129] and eliminate replication blockages. Some early proteins also affect the host cell: they modify the cell cycle (e.g., transition to the S phase) [130,131], affect lipid, glutamine and glucose metabolism [132], which is important for virion membrane production, and inhibit the host’s immune response [108].

In addition, early proteins prepare the conditions for the expression of late genes, i.e., the production of viral structural proteins. Only after viral DNA replication are late genes activated [133,134]. The early phase therefore constitutes the “preparation of the field” for virion production. Understanding this phase is important for both basic research and potential therapeutic strategies (e.g., the “kick and kill” strategy, activation of the virus for elimination) [117,135].

### 2.4. Late Phase (L)

The chromatin of the viral genome in the late phase is usually more decondensed, without typical histone and nucleosome modifications, which promotes active transcription of large amounts of late mRNA [23,136]. Cellular and viral factors supporting the transition to the late phase include: activation of IE and E genes, epigenetic changes (e.g., histone acetylation), nucleotide availability, host cell cycle status, and environmental conditions conducive to virus production (e.g., activated B lymphocytes). The late phase begins after the start of viral genome replication, when viral DNA has been copied in large quantities and the host cell membrane is available for particle assembly [37,136].

The expression of late-phase proteins is strongly dependent on viral DNA replication and often on the formation of the viral genome in a decondensed form (without chromatin) [136,137,138]. The products of these genes are capsid and tegument proteins, envelope glycoproteins needed for the virus to exit the cell and infect other cells, as well as proteins necessary for packaging viral DNA, virion assembly and release [138,139]. Release from the cell occurs via exocytosis or lysis of the host cell [139]. Some late proteins also have functions that help evade the host’s immune system, e.g., through surface glycoproteins that modify antigen presentation, proteins that reduce MHC (major histocompatibility complex) expression, or proteins that modulate immune cells [140].

The expression of late genes is a marker of productive viral infection, which distinguishes it from latency or early activation alone. In the context of EBV-related diseases, such as nasopharyngeal carcinoma or EBV-positive gastric carcinoma, the detection of late genes may indicate active viral replication and may have prognostic or therapeutic significance [141]. Viral DNA polymerase inhibitors, e.g., acyclovir, may act at this stage by blocking DNA replication and, consequently, late gene expression, thereby limiting virus production, but it acts only on the active DNA replication stage of EBV and does not affect the early or partial forms of viral reactivation. Understanding the mechanism of late-phase regulation (e.g., the vPic (viral Pre-Initiation Complex) mechanism [137]) offers potential targets for drugs. If virion production can be completely blocked, the spread of the virus can be inhibited. However, it should be remembered that the malfunctioning of EBV-infected cells is due not only to the presence of whole-virus virions, but primarily to the presence of IE- and E-phase proteins in the cell, which significantly dysregulate cell metabolism. Therefore, dysfunction and dysregulation will already be observed at the IE- and E-phase stages. At this stage, however, replication may stop, as discussed in this article, which can lead to significant disease symptoms in the absence of viral DNA in the plasma.

## 3. General Regulatory Model of EBV Infection

The mechanisms of interaction between viral proteins and the host proteome and genome involve hundreds of different interactions. This publication is an attempt to present a simplified model that will allow for a better understanding of the problem, as well as the development of therapeutic strategies that must be different in various states of chronic infection excitation.

Epstein–Barr virus persists in human hosts by navigating a tightly regulated continuum of transcriptional programs, ranging from deep latency to fully productive viral replication. Rather than existing as discrete states, these programs form an interconnected regulatory network defined by feedback interactions among latent (LAT), immediate-early (IE), early (E), and late (L) phases. The transitions between these phases are controlled by molecular circuits involving viral transcription factors, cellular signaling pathways, epigenetic modifiers, and immune-mediated pressures. The resulting architecture functions as a set of reciprocal and feed-forward loops that, according to the rules of control theory, maintain a dynamic balance between viral quiescence, partial reactivation, and lytic replication. Understanding these interactions is essential for explaining both the stability of lifelong latency and the occurrence of partial lytic activation in physiological and pathological contexts.

## 4. LAT ↔ IE Balance

The transition from the latent to the IE state is the starting point for virus activation in the cell. This state depends on the activation of the first two proteins, Zta and Rta, which further activate the lytic cascade in a positive feedback mechanism. A detailed analysis of the molecular factors that induce and inhibit this stage of induction on the molecular level is presented in the work of Wang et al. [33]. The current work focuses rather on the aspects of the feedback loops regulating this excitation, as well as on certain clinical aspects facilitating this activation, as this is key to understanding why some people experience chronic virus excitation.

### 4.1. Epigenetic and Chromatin-Based Repression of Immediate-Early Gene Expression

In latently infected cells, expression of the immediate-early genes *BZLF1* and *BRLF1* is subject to particularly stringent epigenetic and chromatin-based repression [33]. The viral genome is packaged into nucleosomes and decorated with repressive histone modifications, including histone deacetylation and methylation marks associated with transcriptional silencing. In addition, SUMO- and PARP-dependent mechanisms contribute to the maintenance of latency by stabilizing repressor complexes at viral promoters [47,142]. Cellular factors such as PML (promyelocytic leukemia) nuclear bodies and the DAXX–ATRX complex (death domain-associated protein 6 and alpha-thalassemia mental retardation syndrome X-linked complex) further reinforce this repressive chromatin state by promoting histone loading and limiting the access of transcriptional machinery to immediate-early promoters [143,144,145]. As a result, transcription of *BZLF1* and *BRLF1* represents a high-threshold event that requires coordinated relief of multiple layers of repression.

The likelihood of successful immediate-early gene activation differs across latency programs. In latency 0, which is characterized by near-complete transcriptional silence of viral protein-coding genes, repression is largely epigenetic and therefore potentially reversible in response to strong cellular stress or inflammatory signaling. In contrast, higher latency programs (latency I, II, and III) involve active expression of latent viral proteins and non-coding RNAs that impose additional inhibitory constraints on lytic reactivation [47,48,49,50,51,52,53,146,147]. Latent proteins can directly or indirectly suppress immediate-early gene transcription by modulating host signaling pathways, interfering with chromatin remodeling, inhibiting interferon responses, and reinforcing epigenetic silencing of lytic promoters. Consequently, although latency II and III are transcriptionally more active overall, they may paradoxically be more resistant to spontaneous lytic entry than latency 0. This layered repression ensures that EBV reactivation remains rare and tightly controlled, occurring only in cells in which epigenetic barriers are sufficiently relaxed and activating signals exceed a critical threshold.

### 4.2. Early Phase Proteins Support Lat → IE Transition

Once IE genes are active, early (E) proteins amplify this shift through a second tier of positive feedback that directly or functionally antagonizes HDAC, SUMO, PARP1 and methylation-based repression. The conserved herpesvirus kinase BGLF4 is a central element of this module. BGLF4 phosphorylates multiple chromatin-associated substrates, including histones and chromatin modifiers such as TIP60 (Tat-interacting protein of 60 kDa), and promotes TIP60-dependent histone acetylation in the context of a DNA damage response (DDR) that favors viral replication [118,148,149]. Activation of the TIP60 histone acetyltransferase leads to increased acetylation of histones at viral lytic promoters, functionally opposing HDAC1/2-mediated deacetylation and loosening viral chromatin. Although HDAC proteins remain present, their repressive impact is outweighed by kinase-driven recruitment and activation of HAT (histone acetyltransferase) activity at lytic loci, thus shifting the balance toward an open, transcriptionally active configuration.

The early antigen EA-D, encoded by *BMRF1*, further reinforces this chromatin opening by repurposing a classically repressive complex. BMRF1 interacts directly with the Mi-2/NuRD complex, which in many cellular contexts acts as a HDAC-containing chromatin repressor. During EBV lytic infection, however, BMRF1–NuRD complexes are required for transcriptional activation of viral genes and for the inhibition of canonical double-strand break signaling [119]. In other words, BMRF1 converts NuRD from a latency-supporting repressor into a lytic-supporting activator, thereby functionally neutralizing a DNA methylation-coupled, HDAC-based silencing mechanism and turning it into part of a positive feedback loop that sustains IE/E expression.

The early nuclease/exonuclease BGLF5 contributes to this positive feedback at the level of host gene expression rather than by direct enzymatic modification of chromatin. BGLF5 mediates extensive host shutoff by degrading cellular mRNAs, including those encoding antiviral effectors and components of interferon and immune signaling pathways [150,151,152]. This broad mRNA degradation limits the synthesis of host restriction factors, chromatin regulators, and immune effectors that would otherwise restore a latent-like chromatin state or eliminate lytically active cells. Although BGLF5 does not directly de-SUMOylate or demethylate DNA, its host shutoff activity removes or attenuates many of the cellular systems that enforce EBV chromatin compaction and antiviral defenses, thereby indirectly reinforcing the chromatin-loosening effects initiated by BZLF1 and BGLF4.

PARP1 normally helps maintain EBV latency by binding the *BZLF1* (Zp) promoter and stabilizing a repressive chromatin structure that prevents lytic gene activation. During the early stages of reactivation, however, the chromatin loops and CTCF-dependent domains that depend on PARP1 begin to reorganize, and PARP1 itself is pulled away from the Zp promoter and recruited into newly forming viral replication compartments [103]. Once PARP1 leaves the promoter, it can no longer enforce repression at the lytic switch. This loss of PARP1–CTCF-mediated control makes the chromatin more permissive and helps push the cell toward lytic replication. As BGLF4 and other early viral proteins induce a DDR-like environment and build replication factories, PARP1 and CTCF are increasingly displaced from latent promoters, further weakening the repression systems that normally keep EBV silent.

Taken together, these processes define a coherent positive-feedback architecture at the chromatin level. In the latent state, HDAC1/2, SUMO-organized PML/DAXX/ATRX complexes, PARP1-coordinated chromatin loops and DNA methylation plus MBD-NuRD-type repressors maintain a compact, transcriptionally silent viral episome. When IE proteins like BZLF1 break through this barrier, they do not merely bypass it: they recruit HAT activity, exploit DNA methylation to favor their own binding, and actively dismantle SUMO-dependent repressors. Early proteins then extend and stabilize this opening by converting NuRD into a transcriptional co-activator, activating TIP60-driven acetylation, shutting off host restriction factor synthesis, and reprogramming PARP1’s chromatin functions. Late tegument factors such as BNRF1 [153] complete the process by disassembling DAXX/ATRX/PML-mediated H3.3 loading. The net effect is that once a threshold of IE/E expression is reached, the lytic program feeds back positively on HDAC, SUMO, PARP1 and methylation-based repression, progressively relaxing viral chromatin and making continued lytic gene expression more probable than a return to latency. This architecture explains why EBV can remain stably latent for long periods yet, once sufficiently reactivated, can rapidly commit to a full lytic cycle and also why intermediate, partially lytic states arise when these positive feedbacks are engaged only incompletely or are reined in by immune surveillance.

### 4.3. Positive Couplings Between BZLF1/BRLF1 and AP-1/p38/ERK/JNK Pathways

Activation of the Epstein–Barr virus immediate-early genes *BZLF1* and *BRLF1* is tightly coupled to host stress and inflammatory signaling pathways, forming multiple positive feedback loops that stabilize early lytic gene expression. Cellular pathways such as AP-1, p38 MAPK, ERK, and PI3K/AKT contribute to the initial activation of *BZLF1* and, to a lesser extent, *BRLF1* by integrating signals derived from inflammation, cellular stress, and receptor-mediated stimulation [32,34,154]. Once expressed, the immediate-early proteins ZEBRA (BZLF1) and Rta (BRLF1) reciprocally enhance the activity of these same pathways by inducing MAPK signaling, activating AP-1 transcription factors, and promoting pro-survival PI3K/AKT signaling [117,155,156,157,158]. This bidirectional coupling creates self-reinforcing signaling circuits that amplify and maintain immediate-early and early lytic transcription—even in the absence of sustained upstream stimuli. Importantly, these positive feedback loops lower the threshold for repeated or prolonged early lytic activation while remaining insufficient on their own to trigger viral DNA replication and late gene expression. As a result, EBV can repeatedly exit latency at the transcriptional level and stabilize abortive or partial lytic states, gaining access to viral regulatory functions and host signaling rewiring without crossing the high-threshold checkpoint associated with productive replication and immune-mediated elimination.

### 4.4. Late Phase Supports Lat → IE Transition

Positive feedback facilitating virus excitation and blocking the immune system extends into the late phase, in which many early phase proteins are no longer expressed, but late-phase proteins also contribute to a lesser extent to strengthening the feedback loop that enhances reactivation. Late tegument proteins participate in dismantling SUMO- and PML-dependent chromatin restriction as the lytic program approaches completion. The major tegument protein BNRF1 localizes to PML nuclear bodies and binds the histone H3.3 chaperone DAXX, displacing ATRX from the DAXX–H3.3 complex and preventing deposition of repressive H3.3-rich chromatin on the EBV genome [153]. Because PML nuclear bodies are SUMO-organized hubs of intrinsic antiviral repression, BNRF1-mediated disruption of DAXX–ATRX and reprogramming of H3.3 loading convert a strongly repressive SUMO/PML module into a state permissive for viral gene expression and replication. This action complements the effects of IE and E proteins on HDAC and DNA methylation, further locking the system into a lytic-favoring chromatin configuration.

## 5. Clinical Conditions for the LAT → IE/E Phase Induction

Molecular biology focuses on identifying the molecular factors that awaken cells from a latent state. Below are four clinical conditions in patients that are suspected of having the ability to awaken cells to active lytic activity and for which there are molecular indications that such excitation may occur. In particular, the fact that these are chronic conditions increases the likelihood of chronic activation of lytic activity in the body, which in turn can lead to both severe courses such as CAEBV and mild courses of activation that lead to the development of autoimmune diseases. Table 2 summarizes cellular and systemic factors influencing the probability of the transition to the lytic state.

### 5.1. Parasitic Infections

Parasitic infections are proposed as potential modulators of EBV latency [32,159]. Anti-parasitic immune responses are typically dominated by Th2-associated cytokines such as IL-4 and IL-13, which antagonize Th1-type antiviral immunity and can suppress autophagy [160]. Because autophagy contributes to intracellular pathogen control, long-term Th2 polarization may create a cellular environment that favors persistence and episodic reactivation of latent viruses. The Th2 response modulated by IL-4 and IL-13 also inhibits the activity of CD4^+^ and CD8^+^ cells through a number of mechanisms described in the reviews by Henry et al. [161] and Kumar et al. [162]. The inhibitory effect also occurs via IL-10 in response to Th2, which is additionally mimicked by the BCRF1 virus protein. Although direct evidence linking parasitic infections to EBV reactivation in vivo remains limited, this immunological framework provides a plausible mechanistic connection.

### 5.2. Psychological Stress

Psychological stress represents another well-documented trigger of herpesvirus reactivation [31,163,164,165,166,167,168]. Chronic or intense emotional stress alters immune regulation through neuroendocrine mediators, particularly glucocorticoids, which increase the *BZLF1* transcription [169]. Importantly, transient inflammatory or stress signals may induce partial or rapidly controlled reactivation events, whereas sustained exposure to pro-inflammatory or immunomodulatory stimuli is more likely to promote recurrent or persistent viral activity.

### 5.3. Coexisting EBV and Other Viral Pathogens

EBV frequently coexists with other chronic infections, and accumulating evidence suggests that co-infecting pathogens may facilitate mutual reactivation through shared inflammatory signaling. Viruses such as cytomegalovirus, HSV-1, HHV-6, hepatitis viruses, HIV, and HPV, as well as bacterial pathogens including *Helicobacter pylori*, *Streptococcus* species, and *Aggregatibacter actinomycetemcomitans*, have all been reported to promote EBV reactivation in experimental or clinical contexts [32]. If several intracellular pathogens are present simultaneously, they may exacerbate the body’s inflammatory response and increase the risk of complications [170,171,172,173,174]. Acute infections of various origins may exert similar effects by transiently activating MAPK (p38, JNK, ERK) and stress-responsive pathways that stimulate IE gene expression. Autophagy inhibition is the second element that can contribute to the common existence of different chronic intracellular pathogens [175,176,177,178,179], as many of them are able to inhibit autophagy, thus contributing to a higher inflammatory level. Finally, the mentioned chronic viruses are also able to inhibit interferon activity by their own tegument proteins [143,180,181,182,183,184], thus facilitating the chronic inhibition of immunological function and chronic stimulation of the Lat → IE transition.

### 5.4. Gut Microbiota as a Potential Regulator of the Lat → IE Transition

The intestinal mucosa represents the largest immunological interface in the human body, continuously exposed to trillions of commensal microorganisms and dietary antigens. Under physiological conditions, this interface is maintained in a tightly regulated state of controlled tolerance, in which microbial recognition through pattern recognition receptors (PRRs) is balanced by regulatory T cell (Treg) activity and anti-inflammatory cytokines [185,186]. Short-chain fatty acids (SCFAs), particularly butyrate produced by obligate anaerobic commensals, reinforce epithelial barrier integrity, promote Treg differentiation, and limit excessive inflammatory signaling [187]. Disruption of microbial composition (dysbiosis), increased intestinal permeability, or persistent enteric infections disturb this equilibrium. In such conditions, bacterial components enter the systemic circulation, inducing low-grade endotoxemia and sustained production of IL-6, IL-1β, TNF-α, and IL-23, which reshapes systemic antiviral surveillance [188,189,190].

One of the most reproducible consequences of intestinal barrier disruption and microbial translocation is a shift in CD4^+^ T cell differentiation toward a Th17 phenotype [188,191,192]. Loss of SCFA-producing commensals reduces Treg support and weakens regulatory control, further biasing the immune balance toward IL-6/IL-23-dependent Th17 responses [189,193]. In chronic dysbiosis, this skewing results in sustained IL-17A/F production and persistent activation of NF-κB, MAPK and ERK signaling pathways in multiple tissues [194] which can promote transcriptional permissiveness at lytic promoters, particularly *BZLF1*. Gut-derived inflammatory mediators, including IL-1β, IL-6, and TNF-α, directly stimulate these pathways in circulating immune cells and tissue-resident B lymphocytes [195,196]. Chronic exposure to IL-6, a hallmark of dysbiosis-associated inflammation, promotes sustained STAT3 activation in T cells and has been linked to increased expression of inhibitory receptors such as PD-1 (programmed cell death protein 1). In the context of persistent antigenic stimulation, this signaling environment contributes to the functional exhaustion of CD8^+^ T cells, characterized by reduced interferon-γ production, diminished cytotoxic activity, and impaired viral clearance [197,198]. Such partial loss of effector function may allow repeated early lytic activation of EBV-infected cells without complete immune elimination, thereby stabilizing abortive or partial lytic states.

Chronic low-grade intestine-derived endotoxemia may result in sustained TLR4 signaling and activation of MyD88-dependent (myeloid differentiation primary response 88) cascades, leading to NF-κB (nuclear factor kappa-light-chain-enhancer of activated B cells) nuclear translocation and enhanced AP-1 activity [199,200,201]. Because *BZLF1* and *BRLF1* promoters contain response elements influenced by these transcription factors, prolonged inflammatory signaling may lower the activation threshold for the LAT → IE transition. In this context, dysbiosis acts as a systemic amplifier of signaling pathways already known to support EBV reactivation. Moreover, activation of mTOR (mechanistic target of rapamycin) signaling in chronic inflammatory contexts suppresses autophagy [175], an important intrinsic antiviral mechanism [176] which may facilitate persistence of IE/E-expressing cells. This creates a permissive intracellular environment in which early lytic transcription can proceed without efficient silencing.

Within the framework of chronic partial lytic reactivation, these microbiota-driven processes primarily act on the first two required conditions: persistent LAT → IE stimulation and incomplete immune silencing of early lytic states. When combined with limited but recurrent progression of a small cellular fraction to the late phase, such systemic immune remodeling may sustain long-term viral activity without overt viremia. Thus, intestinal dysbiosis should be considered capable of shifting the LAT/IE equilibrium toward repeated reactivation attempts and contributing to chronic inflammatory states associated with EBV persistence.

### 5.5. Immunosuppression and Clinical Contexts Associated with Increased EBV Reactivation

EBV reactivation is strongly influenced by the immune status of the host and is markedly increased in conditions associated with impaired cellular immunity. In particular, iatrogenic immunosuppression, such as that used in solid organ transplantation, hematopoietic stem cell transplantation, and the treatment of autoimmune diseases, substantially elevates the risk of viral reactivation [202,203,204]. These settings are characterized by reduced CD8^+^ T cell and NK cell surveillance, which are critical for controlling lytically infected cells, thereby permitting expansion of cells undergoing lytic or abortive lytic activation [23,203].

Clinically, EBV reactivation is well documented in transplant recipients, where it may lead to post-transplant lymphoproliferative disorders (PTLDs) [202,204], as well as in patients receiving B cell-depleting therapies such as anti-CD20 antibodies (e.g., rituximab), or other immunosuppressive regimens [32,205,206]. Increased risk has also been observed in patients with chronic kidney disease, particularly those undergoing dialysis or receiving immunosuppressive therapy in nephrology settings [207,208,209].

From a mechanistic perspective, these conditions do not necessarily increase the intrinsic probability of initiating lytic activation at the cellular level, but rather reduce the efficiency of immune-mediated clearance of cells entering lytic or pre-lytic states. Within the framework proposed here, this shifts the system toward a higher steady-state burden of cells in IE/E or partially lytic states, thereby increasing the likelihood of detectable reactivation and viral shedding. These observations have important clinical implications, suggesting that selected patient populations, particularly transplant recipients, individuals receiving B cell-depleting or long-term immunosuppressive therapies, and patients with advanced immunodeficiency, may benefit from continuous monitoring of EBV load and reactivation markers [202,209].

## 6. Regulation of EBV DNA Replication as a Central Checkpoint of Lytic Progression

Many studies have shown that the expression of EBV late genes requires active viral DNA replication. Conditions in which DNA replication is inhibited often block the expression of late genes [136,137,138,210,211]. The study by Aubry et al. showed that EBV late genes require a special viral preinitiation complex (vPIC) composed of viral proteins, which functions only after DNA replication and enables the transcription of late genes [136,137].

Initiation of EBV DNA replication represents a decisive transition point between the early (E) and late (L) phases of the viral cycle. Unlike immediate-early and early gene expression, which can be triggered relatively frequently and even transiently, viral DNA replication requires the precise and coordinated convergence of multiple viral, cellular, epigenetic, and metabolic conditions [37,132,134]. Because all of these requirements must be fulfilled simultaneously and in the correct proportions, the initiation of DNA replication constitutes a high-threshold checkpoint [137]. Failure to meet any single requirement is sufficient to halt progression and results in partial or abortive lytic reactivation without virion production.

For EBV DNA replication to begin, the viral lytic origin of replication (OriLyt) must be activated [137]. This is not a passive event but a highly regulated process that requires the assembly of a functional viral replisome at the OriLyt. Core viral components include a set of early replication proteins that perform distinct but interdependent roles. These include BMRF1 (EA-D) [128,212], which stabilizes the replication complex; BALF2 [128,212], the single-stranded DNA-binding protein; and the viral helicase–primase complex composed of BBLF4, BSLF1, and BBLF2/3. Additionally, BZLF1 protein is an *oriLyt*-binding protein and the BALF5 protein is a DNA polymerase (Pol).

The presence of these proteins alone, however, is not sufficient. They must be expressed at adequate levels, within a narrow temporal window, and assemble correctly at the OriLyt. Classical studies demonstrated that many cells expressing Zta and other early proteins nevertheless fail to initiate DNA replication, underscoring that OriLyt activation is regulated independently of early gene transcription [213].

A critical layer of control lies at the level of chromatin. OriLyt must undergo extensive chromatin remodeling before it becomes replication competent. This involves local histone acetylation, displacement of HDAC-containing repressor complexes, reorganization of DNA methylation-dependent silencers, and relief of SUMO-dependent repression mediated by PML nuclear bodies and the DAXX–ATRX complex [214,215].

BZLF1 (Zta) can preferentially recognize and bind a subset of CpG-methylated Zta-responsive elements (meZREs), indicating that CpG methylation is not uniformly inhibitory for EBV lytic activation but can be “read” by Zta to promote transcription from selected viral regulatory regions [101,216,217]. However, single-cell analyses show that many cells can enter an IE/E transcriptional program without initiating EBV DNA replication, consistent with a frequent arrest at the E–L boundary where origin activation and chromatin remodeling at replication-associated regions (including OriLyt) fail to reach a replication-competent state [101,218,219].

PARP1-dependent chromatin structures contribute to the stabilization of lytic repression by restricting activation of the *BZLF1* promoter, and experimental depletion or pharmacological inhibition of PARP1 results in enhanced EBV lytic progression and viral DNA replication, supporting a gatekeeper role for PARP1 at the transition toward productive replication [220].

Beyond chromatin accessibility, EBV DNA replication depends on the activation of a DNA damage response (DDR)-like signaling cascade, particularly the ATR–Chk1 pathway. Unlike cellular DNA replication, viral replication is initiated in a context that resembles replication stress rather than classical DNA damage. ATR activation promotes the recruitment of replication factors, stabilizes replication forks, and enables the formation of viral replication compartments. Inhibition or knockdown of ATR abolishes EBV DNA synthesis despite intact early gene expression, clearly separating early transcription from replication competence [128]. Importantly, not all cellular stresses generate the appropriate DDR signal for EBV. Partial or improperly configured DDR activation is common and frequently insufficient to support viral DNA replication [221,222,223,224].

Metabolic readiness of the host cell constitutes an additional and often underappreciated constraint. Viral DNA replication is highly energy-intensive and requires abundant nucleotide pools, active biosynthetic pathways, and suppression of catabolic stress responses such as excessive autophagy. Memory B cells and differentiated epithelial cells, two major EBV reservoirs, are frequently metabolically quiescent, rendering them poorly suited to sustain large-scale viral DNA synthesis. Reviews of herpesvirus latency emphasize that metabolic insufficiency alone can arrest reactivation after early gene expression, even when all viral factors are present [225].

Because all the requirements of OriLyt activation, epigenetic remodeling, DDR engagement, replication compartment formation, stoichiometric assembly of replication proteins, and metabolic support must be met simultaneously, EBV DNA replication is intrinsically fragile. Partial fulfillment of these conditions leads to a state in which early lytic genes are expressed but viral genomes are not amplified. This has been directly demonstrated at the single-cell level, where large fractions of reactivated cells exhibit IE and early transcription without any detectable increase in EBV DNA copy number [218]. These cells define the partial or abortive lytic state.

Finally, immune selection strongly reinforces this checkpoint. Cells that successfully initiate EBV DNA replication rapidly increase viral antigen load and become highly visible to cytotoxic T lymphocytes and natural killer cells [152,226,227]. In contrast, cells that remain latent or stall before DNA replication express fewer immunogenic antigens and are far more likely to survive. Over time, immune pressure therefore selectively eliminates fully replicating cells while sparing latent and non-replicating early lytic cells. This selective pressure stabilizes a population of infected cells that either remain latent or repeatedly enter and exit early lytic transcription without crossing the functional replication threshold. Such immune-driven selection provides a powerful explanation for why partial lytic reactivation is commonly observed in vivo and why EBV persistence is dominated by non-replicating states despite frequent molecular signs of reactivation.

Together, these observations support a model in which EBV DNA replication functions as a central regulatory bottleneck. Its initiation requires the synchronized convergence of multiple rare conditions, and its failure represents the most common outcome of reactivation attempts. This checkpoint not only limits viral production but also shapes the long-term equilibrium between the virus and the host’s immune system, allowing EBV to persist for life while minimizing immunopathology.

## 7. Abortive/Partial Lytic Reactivation as a Common Outcome of EBV Reactivation

For many years, EBV lytic reactivation was conceptualized as a binary process, in which latently infected cells either remained silent or entered a full productive lytic cycle culminating in viral DNA replication, late gene expression, and virion production. However, accumulating evidence from transcriptional, single-cell, and functional studies demonstrates that this dichotomous view is overly simplistic. Instead, partial lytic reactivation emerges as a frequent and biologically relevant outcome, both during primary infection and during reactivation from latency.

### 7.1. Partial Lytic Gene Expression During Early EBV Infection and Latency Establishment

Direct experimental evidence that partial lytic reactivation accompanies the establishment of latency was provided by Inagaki et al., who combined time-resolved transcriptomics with fate-mapping approaches in newly infected B cells [211]. During the pre-latent phase, infected cells exhibited a transient burst of immediate-early and early lytic gene expression, while infectious virion production remained undetectable. Mathematical modeling integrated with experimental data supported the conclusion that viral DNA replication does not occur at this stage, defining a bona fide partial lytic state rather than delayed productive replication. Importantly, cells that transiently expressed lytic genes subsequently converged into stable latency, demonstrating that partial lytic reactivation can function as a physiological intermediate rather than a dead-end pathway. These findings extend earlier observations that EBV genomes entering B cells are initially unmethylated, allowing limited Zta-driven transcription without enabling the full lytic transcriptional cascade or DNA replication, thereby structurally biasing early infection toward abortive outcomes.

### 7.2. Partial Lytic Reactivation as a Continuum Revealed by Single-Cell Approaches

Single-cell transcriptomic analyses have also fundamentally reshaped the understanding of EBV reactivation heterogeneity. SoRelle et al. demonstrated that reactivation does not produce discrete latent versus lytic populations, but rather a continuum of cellular states ranging from partial to fully productive reactivation [218]. Cells expressing BZLF1 frequently failed to progress to late gene expression, even under strong lytic induction, and instead adopted distinct transcriptional programs characterized by NF-κB and IRF3 activation. Critically, these partial states were not rare outliers but constituted a substantial fraction of reactivating cells, indicating that failure to complete the lytic cascade is an intrinsic and recurrent feature of EBV biology. This heterogeneity persisted across multiple B cell models, reinforcing the generalizability of partial lytic reactivation as a common outcome.

### 7.3. Partial Lytic States in Epithelial Infection and Tumor-Associated EBV

Evidence for frequent partial or incomplete lytic reactivation is not limited to B cells. Studies of epithelial-tropic EBV strains and EBV-associated malignancies further support the prevalence of partial lytic programs. Tsai et al. [228] described EBV strains displaying spontaneous lytic gene expression without proportional virion production, particularly in epithelial contexts. This decoupling of early/late gene expression from productive replication highlights strain- and cell-type-dependent constraints on lytic completion. Consistently, transcriptomic surveys of EBV-positive tumors reveal the expression of immediate-early and early lytic genes, often accompanied by low-level or “leaky” late gene transcription, while overt viral replication remains absent. Comprehensive synthesis of these observations has been provided by Yap et al. [229], who emphasize that partial lytic gene expression is widespread in EBV-associated cancers and likely contributes to pathogenesis without requiring virion production.

### 7.4. Early Lytic Reactivation and Host Shutoff Without Late-Phase Completion

Mechanistic insight into early partial lytic states has been further refined by studies dissecting host transcriptional remodeling. Buschle et al. [226] showed that induction of the lytic program triggers global chromatin reorganization and host transcriptome repression prior to viral DNA replication, even in systems incapable of entering the late phase. These findings indicate that profound cellular reprogramming can occur during early lytic stages independently of productive replication. More recently, Casco et al. [152] employed dual-fluorescent reporter EBV to isolate early and late lytic populations and demonstrated that extensive host shutoff already occurs during early lytic reactivation, with only a minority of cells progressing to the late phase. This approach provided quantitative confirmation that limited early lytic states dominate reactivation events even under stimulatory conditions.

Collectively, these studies converge on a unifying conclusion: partial lytic reactivation is not an exception but a frequent, perhaps dominant, mode of EBV reactivation. Immediate-early and early lytic gene expression can occur transiently or persistently without completion of the full lytic cascade, without viral DNA amplification, and without virion production. Such states are observed during primary infection, latency maintenance, reactivation from latency, and within EBV-associated tumors.

This recognition provides a conceptual foundation for the subsequent analysis of dynamic equilibria between latent, IE/E-expressing, and late-phase cells. In particular, it supports the hypothesis that immune surveillance, cellular constraints, and viral regulatory mechanisms collectively shape a steady-state distribution dominated by partial lytic outcomes. These considerations naturally motivate the application of mathematical and system-level models to describe how repeated, incomplete reactivation events can sustain long-term viral persistence.

## 8. Conditions Necessary for the Chronic Partial Lytic Reactivation State

A persistent state of chronic partial lytic reactivation, conceptually related to the extreme phenotype observed in chronic active EBV (CAEBV), reflects a dynamic balance between viral reactivation, immune control and maintenance of the latent reservoir. Within the conceptual framework proposed here, three functional conditions can be distinguished that together may support long-term viral persistence. First, there must be sustained or recurrent induction of the latent-to-immediate-early (LAT → IE) transition. The possible drivers are postulated to be chronic inflammatory signals, stress responses, co-infections (chronic viruses and parasites), or other epigenetic relaxation factors. Such repeated stimulation could maintain a continuous influx of cells entering immediate-early and early transcriptional programs. Second, both intracellular antiviral mechanisms (including interferon signaling, chromatin-based repression, autophagy, and intrinsic restriction factors) and systemic immune responses (notably CD8^+^ cytotoxic T cells and NK cells) should be insufficient to fully extinguish IE/E activation, yet still effective enough to prevent widespread productive replication. This partial immune control would stabilize cells in early lytic states rather than eliminate them or allow full viral amplification.

The third condition, involving periodic completion of the late phase, is conceptually linked to the need for maintaining and potentially expanding the infected cell pool. In its classical interpretation, productive lytic replication enables the generation of infectious virions capable of infecting new target cells, including both B lymphocytes and epithelial cells. However, this condition requires careful qualification. EBV persistence within the memory B cell compartment does not depend on continuous reinfection, as the viral genome is maintained as a nuclear episome that replicates in synchrony with host cell division. As a result, the latent reservoir can, in principle, be stably maintained through proliferation and differentiation of infected B cells without the need for ongoing production of the infectious virus.

At the same time, productive lytic replication may still play an important, though possibly intermittent, role in the broader ecology of EBV infection. In vivo, full lytic replication appears to occur predominantly in epithelial cells, particularly within the oropharyngeal compartment, where it contributes to viral shedding into saliva and transmission between hosts. Under this view, late-phase completion may be spatially and temporally restricted rather than continuously required for persistence within the B cell compartment.

Taken together, these observations suggest that the third condition should not be viewed as an absolute requirement for the maintenance of EBV persistence. In particular, periodic or low-frequency completion of the lytic cycle may support viral dissemination, infection of new cellular niches, or replenishment of infected cell populations, while not being strictly necessary for the maintenance of the existing latent reservoir.

Importantly, this condition remains largely inferential and requires direct experimental validation. It generates several testable predictions, including the presence of rare events of full late-phase completion in vivo, their contribution to the renewal of infected cell populations, and the detectability of late-phase viral transcripts or proteins in sensitive longitudinal or single-cell analyses. Within this framework, EBV persistence emerges as a dynamic system in which long-term stability can be achieved through multiple, partially redundant mechanisms, including both episomal maintenance in proliferating B cells and intermittent productive reactivation in selected cellular compartments.

## 9. Discussion

### 9.1. EBV Persistence as a Dynamically Stabilized Survival Strategy

Evolution has shaped EBV toward a lifelong persistent infection characterized by predominant latency and only sporadic reactivation. From a viral fitness perspective, excessive lytic activity would increase immune recognition and clearance, whereas too infrequent reactivation would limit transmission. The observed balance between latency and episodic reactivation therefore likely reflects an optimization between persistence and spread.

When EBV-infected B cells enter full lytic replication, they express high levels of viral antigens and become highly visible to the immune system, leading to efficient elimination by CD8^+^ T cells and NK cells [229,230]. Thus, despite multiple immune evasion mechanisms, cells undergoing full lytic replication appear to be effectively controlled.

Liu et al. [231] reported strong CD8^+^ T cell responses against IE/E lytic antigens, commonly interpreted as evidence of the immunodominance of early lytic phases. However, an alternative explanation may be that this pattern reflects the distribution of infection states within the EBV^+^ cell population. If a substantial fraction of infected cells undergoes incomplete lytic reactivation, with expression of IE and some early genes but limited progression to late stages, the apparent immunodominance of IE/E antigens may arise from increased target availability rather than intrinsically stronger immune recognition.

Under this framework, the relatively stable detection of CD4^+^ responses against late antigens, despite the presumed lower frequency of fully lytic cells, may suggest that cells reaching late stages are subject to substantial immune pressure. However, current data based on peptide-specific T cell frequencies do not allow direct inference about the efficiency of immune control at different stages of the lytic cycle.

Testing this hypothesis will require approaches that directly link the lytic state of individual EBV-infected cells with their susceptibility to immune recognition, taking into account antigen presentation and the relative frequency of complete versus incomplete lytic cycles in vivo.

Finally, caution is warranted when extrapolating findings from acute infectious mononucleosis to chronic infection. Acute infection is characterized by strong inflammatory responses and rapid expansion of EBV-specific T cells, whereas chronic infection involves extensive viral modulation of the host immune environment. In particular, early lytic phases are enriched in viral mechanisms that interfere with antigen presentation and immune signaling, which may further shape the apparent hierarchy of immune responses.

### 9.2. Chronic Stability and Biological Significance of Partial Lytic Reactivation

The convergence of subthreshold induction signals, epigenetic repression, immune surveillance, tissue-specific environments, and viral genomic constraints creates conditions in which Epstein–Barr virus frequently enters a stable state of partial lytic reactivation rather than completing productive replication. In this state, EBV gains selective advantages, including modulation of host cell cycle regulation, inflammatory signaling, and pro-survival pathways, without incurring the immunological cost associated with virion production. The single-cell transcriptomic analyses by SoRelle et al. demonstrate that partial lytic expression programs represent distinct and durable cellular states rather than transient intermediates, supporting the concept that EBV occupies a continuum of activation states rather than a simple latency–lytic dichotomy [218]. This view is consistent with epigenetic models of EBV gene regulation, which emphasize that incomplete lytic transcription frequently arises from interactions between the viral genome and host chromatin architecture [225].

A central insight emerging from these studies is that viral DNA replication constitutes a decisive molecular checkpoint during EBV reactivation. Initiation of immediate-early and early lytic gene expression alone does not commit a cell to productive infection. Instead, infected cells diverge at the point at which viral genome amplification would normally begin. Cells that successfully initiate DNA replication undergo global host shutoff, late gene expression, and rapid immune-mediated elimination. In contrast, cells that fail to initiate replication remain viable, transcriptionally active, and metabolically intact, despite having exited latency at the level of early lytic gene expression. Single-cell trajectory analyses reveal that these partial lytic cells form a reproducible and stable branch of the reactivation landscape, characterized by the expression of regulatory and immunomodulatory viral genes in the absence of late structural gene expression or virion production [218].

This branching architecture has important biological and clinical implications. Partial lytic reactivation enables infected cells to persist while continuously shaping their microenvironment. Cells expressing early lytic genes can sustain low-level inflammatory signaling, alter cytokine and interferon responses, and modulate antigen presentation, yet remain below the functional threshold for efficient cytotoxic T cell elimination. In epithelial tissues, particularly in nasopharyngeal carcinoma, partial lytic gene expression has been shown to promote tumor-supportive microenvironments through the secretion of cytokines and chemokines that recruit immunosuppressive myeloid cells and enhance angiogenesis, thereby contributing to oncogenesis rather than viral spread [210]. In B cell-associated diseases, partial lytic reactivation generates a reservoir of long-lived infected cells that repeatedly express immunomodulatory viral proteins, potentially exacerbating immune exhaustion and increasing the risk of lymphoproliferative disorders, especially under conditions of immunosenescence or partial immunosuppression [218].

At the systemic level, persistent partial lytic reactivation provides a plausible framework for understanding the chronic immune activation associated with EBV in the absence of overt viral replication. Partial lytic activity has been suggested directly or indirectly in the tissues and peripheral blood of patients with autoimmune and inflammatory conditions, including multiple sclerosis and systemic lupus erythematosus, often without detectable virion production [8,232,233,234]. Rather than reflecting failed immune control, this pattern is consistent with an evolved equilibrium in which immune surveillance efficiently eliminates cells entering full lytic replication, while selectively sparing cells stalled before DNA replication. Over time, this immune selection enriches partially reactivated states, making partial lytic reactivation a dominant active form of EBV infection in immunocompetent hosts [218].

Taken together, these findings support the view that partial lytic reactivation represents a stable, biologically meaningful, and clinically relevant outcome of EBV reactivation. This state allows EBV to persist, modulate host immunity, and contribute to chronic inflammation and oncogenic risk without triggering the immune responses associated with productive replication. Recognizing partial lytic reactivation as a distinct and durable component of the EBV lifecycle has important implications for biomarker interpretation and for therapeutic strategies aimed at either enforcing complete viral silencing or deliberately driving the virus into a fully lytic, drug-targetable state.

### 9.3. Building the Mathematical Models of Lat/IE/E/L Equilibrium

The mechanisms of dynamic equilibrium between the pool of latent cells, the fraction of cells in the IE/E phases, and the rare fraction reaching the late phase can be formalized using mathematical models based on systems of ordinary differential equations describing flows between states and interactions with the immune response (e.g., Lat → IE/E transitions, arrest/disappearance, transition to late phase, and the production and clearance of free virus). This approach has already been applied in practice in the context of EBV by Inagaki et al., who combined experimental data with in silico simulations within a parameterized mathematical model of infection dynamics and considered scenarios with or without progeny virus production, showing that the model best reproduces the observed dynamics in a variant consistent with partial infection in the pre-latent phase [211]. This direction should be developed and expanded to include further regulatory layers relevant to chronic EBV infection, including explicit consideration of the heterogeneity of IE/E/L states at the single-cell level, cycle phase-dependent immunoevasive mechanisms (especially early ones), and modulation by environmental factors affecting CD4^+^/CD8^+^ efficiency (e.g., chronic psychological stress, parasites, and chronic viruses). At the same time, this approach is consistent with the broader literature on EBV modeling within the host, where differential models have already been proposed to describe the dynamics of the virus, infected cells, and CD8^+^ response (e.g., in the context of age differences and mononucleosis risk), which further supports the thesis of the usefulness of formal models for integrating immunological and virological data in chronic diseases [235] and solving the equilibrium states according to the rules of control theory.

### 9.4. The Challenge of Detecting Partial Lytic Reactivation

Many autoimmune diseases are somehow linked to infection with EBV (multiple sclerosis [1,2,3,4], rheumatoid arthritis [5,6,7], Sjogren’s syndrome [5,7], systemic lupus erythematosus [7,8], fibromyalgia [9,10], Hashimoto thyroiditis [11,12,13], Graves’ disease [11,236], autoimmune hepatitis [237], and hemophagocytic lymphohistiocytosis [14,15]) and studying these links is essential for developing diagnostic and therapeutic strategies. The development of standardized and clinically interpretable assays targeting early lytic transcriptional activity remains a critical unmet need.

Accurate detection of active EBV infection remains a major clinical challenge. Serological testing has limited diagnostic value, as EBV-specific IgG antibodies persist for life after primary infection and do not reflect the current state of viral activity. Conversely, IgM antibodies and early antigen (EA) responses typically disappear within a few months after primary infection and are therefore insensitive markers of chronic or intermittent reactivation [238,239,240]. Similarly, detection of EBV DNA in whole-blood or peripheral blood mononuclear cells (PBMCs) primarily reflects the presence of latently infected B lymphocytes and does not distinguish between latent and active phases of infection.

The measurement of EBV DNA in plasma is more indicative of active lytic replication, as viral DNA enters the circulation following cell lysis and virion release. In advanced or severe disease states, plasma viremia is frequently detectable and correlates with ongoing productive infection. However, this approach fails to capture a large and potentially clinically relevant population of infected cells that enter immediate-early (IE) or early (E) lytic programs but do not progress to the late phase. Cells in partial lytic reactivation may remain viable and do not release virions into plasma.

Since viral DNA is detected in the plasma of 3–8% of the population, this raises questions about the dynamics of this process and to what extent it reflects a complete lytic process versus the killing of cells in a state of partial lytic activation. The second question concerns the type of cells that are the source of viral DNA in plasma. Are these lymphocytes, or perhaps other types of cells, particularly those originating from tissues infiltrated by EBV^+^ lymphocytes?

B lymphocytes and oropharyngeal epithelial cells represent well-established sites of latent infection and productive lytic replication [241]. However, the viruses are released to saliva and not to the blood. EBV DNA and gene expression have also been reported in additional settings, including smooth muscle tumors [242,243] and gastric adenocarcinoma [244]. These observations suggest that EBV may access a broader range of cellular environments. However, their interpretation requires caution. In particular, in many non-lymphoid tissues, EBV-positive signals may reflect the presence of infiltrating infected lymphocytes rather than direct infection of resident parenchymal cells. This distinction is especially relevant in inflamed organs such as the liver, central nervous system, myocardium, and gastrointestinal tract, where EBV-associated pathology has been described, but the precise cellular source of viral signals often remains uncertain.

One possible explanation that may reconcile the observations is that localized reactivation events within EBV-infected lymphocytes generate spatially restricted viral activity in peripheral tissues. In analogy to the oropharyngeal compartment, such events could transiently expose the neighboring cells in different organs to viral gene expression and limited infection. However, it remains unclear whether such processes lead to stable infection of non-classical target cells or instead reflect transient and spatially confined interactions. In this context, it is conceivable that viral gene expression may frequently remain restricted to early or abortive phases of the lytic program rather than progressing to full productive replication. While this interpretation is consistent with the concept of partial lytic reactivation, it should be regarded as a working hypothesis that requires direct experimental validation.

A major challenge in detecting partial lytic reactivation is the probable absence of cell lysis and the consequent lack of circulating viral DNA. In situations where EBV-infected cells undergo lysis or immune-mediated clearance, intracellular viral components may be released into the circulation. Consistent with this, soluble forms of the immediate-early protein ZEBRA (BZLF1) have been detected in the serum of patients with EBV-associated lymphoproliferative disorders [245]. Nevertheless, systematic analyses of other lytic proteins, including early and late gene products, in serum or plasma are currently lacking [246], and the diagnostic relevance of circulating viral protein profiles remains to be established. In particular, quantitative assessment of the relative abundance and proportion of immediate-early, early, and late proteins may help to distinguish between full productive lytic replication and abortive (partial) lytic states, and to estimate the relative contribution of these processes to circulating EBV DNA levels.

By contrast, low-level abortive lytic reactivation may occur, in theory, without cell death and therefore without the release of viral DNA or intracellular proteins into the circulation. Under such conditions, extracellular vesicles, including exosomes, may represent an alternative source of viral biomarkers. EBV-infected cells have been shown to release vesicles containing viral RNA and microRNAs, particularly those derived from the *BHRF1* region, which can be detected in plasma or saliva independently of cell lysis [247,248]. While these findings are promising, the extent to which extracellular vesicle-associated viral components reliably reflect abortive lytic activity in vivo remains to be determined.

When analyzing the problem of EBV reactivation, it should be kept in mind that the pattern of latency interspersed with episodic and often incomplete reactivation is not unique to EBV but reflects a broader biological strategy shared by many persistent human viruses. Other herpesviruses, including human cytomegalovirus [249,250], herpes simplex virus, varicella–zoster virus, HHV-6/7 [225,251], and HIV-1 [252], similarly establish lifelong latency punctuated by transcriptional or early lytic activation that frequently fails to progress to productive replication. In these infections, early viral gene expression can modulate host immunity and tissue environments without detectable viremia, while cells entering full replication are preferentially eliminated by immune surveillance, resulting in dominance of partial reactivation states. Comparable principles apply beyond herpesviruses: latent HIV infection is characterized by frequent transcriptional bursts without virion production, and hepatitis B virus maintains transcriptionally active cccDNA under immune control, with inflammatory flares occurring in the absence of sustained viremia [253,254,255].

Because partial reactivation can drive chronic immune stimulation, cytokine imbalance, and epigenetic remodeling without overt productive infection, similar immunopathological outcomes, including chronic inflammation and autoimmunity, may arise from the reactivation of different latent viruses. This biological convergence substantially complicates correlation-based studies that attempt to link individual viral markers to specific autoimmune diseases, as overlapping immune signatures may reflect partial reactivation of distinct persistent pathogens rather than a unique causal relationship. In this context, partial lytic reactivation should be viewed as an evolutionarily conserved feature of host–virus coexistence rather than an exceptional failure of immune control. This perspective shifts the conceptual focus from binary viral states to dynamically stabilized intermediate configurations governed by regulatory thresholds.

### 9.5. Redefining “Kick and Kill” Strategy

Classical “kick and kill” strategies in EBV-associated diseases are generally conceptualized as induction of the transition from latency to the lytic program, with the implicit assumption that lytic activation renders infected cells susceptible to immune-mediated clearance or antiviral therapy. In this framework, the critical step is typically considered to be the initiation of immediate-early (IE) gene expression.

However, within the framework proposed here, the early lytic state (IE/E) may represent a quasi-stable attractor rather than a transient intermediate. If so, the induction of IE or early lytic genes alone may not be sufficient to achieve effective elimination of infected cells, as a substantial fraction of cells may remain trapped in abortive lytic states that do not progress to stages associated with full immunological visibility or activation of lytic enzymatic machinery.

Under these conditions, two alternative trajectories for exiting the IE/E state can be considered. One possibility is re-silencing, returning to latency and restoring a low-immunogenic state. The second is progression toward a replication-competent lytic program, including late gene expression, which is more likely to expose infected cells to immune recognition and to enable antiviral drug activation.

These considerations suggest that effective therapeutic strategies should not focus solely on inducing the transition from latency to early lytic states, but also on promoting progression beyond the IE/E checkpoint. In this sense, the functional objective of “kick and kill” may need to be redefined from simple lytic induction toward facilitating a transition into therapeutically actionable lytic states (“push-through and kill”).

Moreover, such approaches may need to be combined with interventions that enhance the elimination phase, including restoration of interferon signaling, enhancement of antigen presentation, or activation of autophagy-related pathways. Together, these mechanisms may increase the susceptibility of EBV-positive cells that have been driven out of latency.

A key challenge for future studies will be to define the molecular and metabolic conditions required for progression from early to late lytic phases. In particular, the requirement for coordinated viral gene expression, chromatin remodeling, DNA replication, and metabolic support suggests that this transition represents a high-threshold checkpoint that may be difficult to overcome therapeutically without targeted modulation.

### 9.6. Possible Reversal from Partial Lytic to Latent State

Assuming that partial lytic reactivation is a quasi-stable metabolic state within the cell, another potential therapeutic strategy might be to attempt to return the cell to the latent phase. Although direct evidence for such a transition remains limited, several lines of evidence suggest that such a reversal may be biologically plausible. In particular, strong latency-maintaining mechanisms may not only prevent progression toward productive replication, but may also actively promote re-establishment of a latent-like state following incomplete reactivation.

Importantly, type I interferon signaling represents a key inhibitory axis at the level of the latency-to-lytic switch. Interferon-mediated activation of the JAK–STAT pathway suppresses *BZLF1* expression and EBV reactivation, thereby stabilizing latency [123]. Within the framework proposed here, this suggests that interferon signaling may not only inhibit the Lat → IE transition, but may also functionally bias cells undergoing abortive lytic activation toward the re-silencing of viral gene expression and return to a latency-like state.

In addition, cellular transcriptional programs associated with proliferation and differentiation appear to counteract lytic reactivation. The c-Myc/E2F1 axis has been shown to suppress spontaneous or “leaky” expression of *BZLF1* and to stabilize latency [256]. Sustained activity of this axis may therefore represent a mechanism by which cells resist or reverse early lytic activation. From a therapeutic perspective, modulation of this axis, either by maintaining proliferative signaling or by preventing its collapse, could contribute to limiting abortive lytic activity and promoting latency re-establishment.

Similarly, retinoic acid receptor (RAR/RXR) signaling has been reported to inhibit BZLF1-mediated transactivation and suppress EBV reactivation [257]. Pharmacological activation of this pathway may therefore represent an additional mechanism to restrain early lytic gene expression. Although these approaches contrast with classical “kick and kill” strategies, they may be particularly relevant in contexts where chronic partial lytic activity contributes to pathogenesis. In such settings, therapeutic strategies aimed at reinforcing latency or promoting re-silencing of partially activated lytic programs may represent a rational alternative.

## Figures and Tables

**Table 2:** Cellular and systemic factors regulating transitions within the EBV lytic program. The table summarizes key pathways and environmental influences that modulate transitions between latency (L), early lytic activation (IE/E), and productive late replication (late). Several signaling pathways, including p38 MAPK, ERK, AP-1, and ROS-associated stress responses, converge on activation of immediate-early genes such as BZLF1, promoting the L → IE transition. In contrast, progression to productive lytic replication depends on additional requirements, including ATR-dependent DNA damage response, highlighting a higher regulatory threshold for the E → late transition. Other factors, such as PI3K/AKT signaling, autophagy, immune context, and systemic influences (e.g., psychological stress), modulate the outcome of reactivation by affecting cell survival, immune control, and the likelihood of progression from the partial lytic state.

Factor	Mechanism	Effect	Transition
p38 MAPK	AP-1 activation → *BZLF1*	Promotes IE gene expression	L → IE
ERK	AP-1 activation → *BZLF1*	Promotes IE gene expression	L → IE
PI3K/AKT	Survival and metabolic signaling	Supports cell survival during lytic activation	Context dependent
AP-1	Transcriptional activation	Induces IE genes	L → IE
ROS	Stress signaling → AP-1	Promotes IE expression	L → IE
ATR/DDR	Replication stress response, support of viral replication compartments	Enables viral DNA replication	E → Late
Macrophage polarization (M1/M2)	Cytokine milieu (e.g., IL-10 vs. proinflammatory signals)	Modulates immune clearance of infected cells	System–level (immune control)
Autophagy	Flux modulation by EBV (induction with impaired completion)	Modulates lytic progression and immune recognition	System-level
Psychological stress	HPA axis activation and immunomodulation	Associated with increased frequency of lytic reactivation	L → IE (indirect)

## Data Availability

No new data were created or analyzed in this study. Data sharing is not applicable to this article.
